# Gut Microbiota in Adipose Tissue Dysfunction Induced Cardiovascular Disease: Role as a Metabolic Organ

**DOI:** 10.3389/fendo.2021.749125

**Published:** 2021-09-06

**Authors:** Xinyu Yang, Xianfeng Zhang, Wei Yang, Hang Yu, Qianyan He, Hui Xu, Shihui Li, Zi'ao Shang, Xiaodong Gao, Yan Wang, Qian Tong

**Affiliations:** ^1^Department of Cardiovascular Medicine, First Affiliated Hospital of Jilin University, Changchun, China; ^2^Institute of Materia Medica, Chinese Academy of Medical Sciences and Peking Union Medical College, Beijing, China; ^3^Department of Neurosurgery, First Affiliated Hospital of Jilin University, Changchun, China; ^4^Department of Neurology, First Affiliated Hospital of Jilin University, Changchun, China

**Keywords:** gut microbiota, adipose tissue dysfunction, molecular endocrinology, cardiovascular disease(s), gut dysbiosis****

## Abstract

The gut microbiome has emerged as a key regulator of host metabolism. Accumulating evidence has indicated that the gut microbiota is involved in the development of various human diseases. This association relies on the structure and metabolites of the gut microbiota. The gut microbiota metabolizes the diet ingested by the host into a series of metabolites, including short chain fatty acids, secondary bile acids, trimethylamine N-oxide, and branched-chain amino acids, which affects the physiological processes of the host by activating numerous signaling pathways. In this review, we first summarize the various mechanisms through which the gut microbiota influences adipose tissue dysfunction and metabolic processes that subsequently cause cardiovascular diseases, highlighting the complex interactions between gut microbes, their metabolites, and the metabolic activity of the host. Furthermore, we investigated the current status of clinical therapies for adipose tissue dysfunction directed at the gut microbiota. Finally, we discuss the challenges that remain to be addressed before this field of research can be translated to everyday clinical practice.

## 1 Introduction

Obesity is a major global health concern. There is a general consensus that obesity increases the risk of cardiovascular diseases (CVDs). By far the most widely accepted measure of obesity is the body mass index (BMI), a simple calculation based on an individual’s weight in kilograms divided by the square of their height in meters (kg/m^2^) (1). A high BMI has been associated with shorter life expectancy (2), which is mainly due to increased risk of type 2 diabetes (T2D), hypertension, dyslipidemia, and CVD (3).The Global Burden of Disease study group estimated that elevated BMI values were responsible for 4 million deaths in 2015, with two thirds of this number attributed to CVDs (4). However, it has been pointed out that the BMI alone cannot fully identify patients at high-risk of CVDs. Wildman et al. (5) found that a substantial proportion, approximately 50% of overweight individuals and 30% of obese individuals, are free from any obvious signs of metabolic or cardiovascular complications. Furthermore, CVDs are also common in people with normal BMI, which suggests BMI is a highly heterogeneous indicator. That is because BMI is an overall indicator related only to height and weight, which does not take body composition into account. At an individual level, BMI can neither distinguish between fat and lean tissue nor determine fat distribution, function, and associated risk factors (1).

Adipose tissue dysfunction (ATD) or “adiposopathy” is a relatively recent concept and is thought to be more closely associated with CVDs than the BMI (6). In the past, the adipose tissue was thought to be relatively inert and its only function was as a storage depot for excess energy in the form of triglycerides and to build up or break down excess lipid into free fatty acids and glycerol based on the metabolic needs of the body. However, growing research suggests that adipose organs are considered to be quite active tissues with metabolic functions and are involved in crosstalk between multiple organ systems (7).

In recent years, a significant number of studies have been conducted investigating the role of gut microbiota in various endocrine and immune diseases, such as obesity, diabetes, nonalcoholic fatty liver disease, allergies, and CVDs (8, 9). There has been much literature summarizing the relationship between gut microbiota and CVDs, involving a wide range of gut microbiota metabolites and their endocrine functions *in vivo (* 10, 11). To date, the gut microbiota is considered a novel endocrine organ that effects many metabolic activities in the host. In this review, we provide a brief review of the gut microbiota and its metabolites, and describe the endocrine roles and molecular mechanisms involving the gut microbiota and metabolites in various aspects of ATD-induced CVDs. Finally, we also briefly review the treatment of various aspects of ATD-induced CVDs targeting the gut microbiota and thus, provide guidance for future research in the emerging field of the gut microbiome associated with the development of metabolic diseases in humans.

## 2 Adipose Tissue Dysfunction

**Figure 1:**
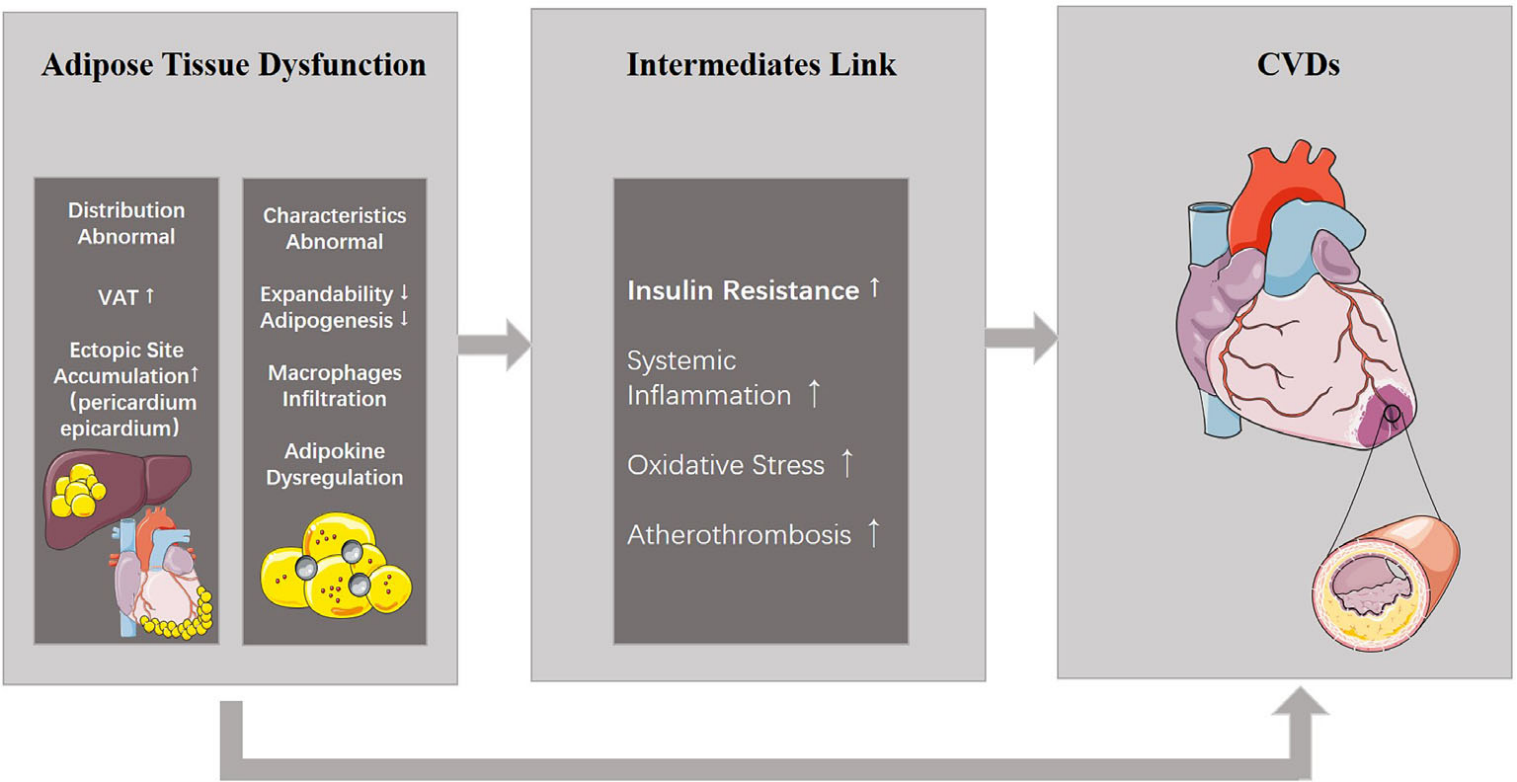
ATD and its relationship with CVDs. ATD consists of two aspects: abnormal distribution and properties. ATD is able to induce CVDs directly, or results in other metabolic diseases including insulin resistance which eventually leads to CVDs. ATD, adipose tissue dysfunction; CVDs, cardiovascular diseases.

This concept of ATD further details the phenotype of obese patients. ATD consists of 2 aspects: abnormalities in fat distribution and abnormalities in the characteristics of adipose tissue, which are both highly related to CVDs (**Figure 1**).

Fat distribution abnormalities refer to the excessive accumulation of visceral adipose tissue (VAT), or fat storage in the intraperitoneal and retroperitoneal spaces, and deposition of ectopic fat, fat stores in body locations where fat is not physiologically stored such as the liver, pancreas, heart, and skeletal muscle. Normally, adipose tissue accumulates in subcutaneous adipose tissue (SAT, 80-90%) (12) and the main depots of SAT are the abdominal, subscapular (on the upper back), and gluteal and femoral areas (13, 14). The SAT depot is located under the skin and does not communicate with internal organs. It is considered as a normal physiological buffer for excess energy intake with little threat to cardiovascular health (15). Conversely, through excessive energy intake, sex hormones levels (16), use of glucocorticoids (17), genetic make-up (18), and epigenetic mechanism (19), fat tends to store in the intraperitoneal and retroperitoneal areas, and in locations where it is not physiologically stored such as the liver, pancreas, heart, and skeletal muscle, which has been defined as VAT. Ross et al. (20) found that individuals matched for abdominal SAT with low or high VAT levels had different levels of glucose tolerance, whereas those matched for VAT had similar glucose tolerance testing with high and low SAT. This study indicated that SAT may not be a risk factor for metabolic diseases, whereas VAT and ectopic fat accumulation was causally related with insulin levels. In addition, several studies have reported positive associations of excess VAT accumulation with cardiovascular risk factors, CVDs and all-cause mortality (21–23). Furthermore, adipose tissue accumulation at special ectopic sites such as the pericardium or epicardium also results in increased CVD risk (24).

Another aspect of ATD involves abnormalities in the characteristics of adipose tissue. Adipocytes typically constitute the majority of the cellular content of adipose tissue. Adipocytes are surrounded by fibrous connective tissue, collagen, nerves, and blood vessels. Despite its location, the metabolic activity of adipose tissue is also a determinant of ATD-induced CVDs. Features of dysfunctional adipose tissue include impaired adipose tissue expandability and adipogenesis (20), as well as hypertrophy and altered lipid metabolism by fat cells. Most importantly, macrophage infiltration can be observed in ATD and initiates a vicious cycle of inflammatory response (25), leading to polarization of macrophages toward a pro-inflammatory phenotype (M1-polarized), which can activate inflammatory pathways and impair insulin signaling (26). In addition, adipokines, the bioactive compounds which are synthesized in the adipose tissue and released into circulation, can be dysregulated in ATD. This dysregulation is a prominent hallmark of ATD (27). Expression of pro-inflammatory adipokines such as leptin, tumor necrosis factor α (TNF-α), interleukin-6(IL-6), interleukin-8(IL-18), retinol binding protein 4, lipocalin 2, and angiopoietin-like protein 2 far exceeds that of anti-inflammatory factors such as adiponectin, IL-10, and nitric oxide (NO). The imbalance between pro- and anti-inflammatory cytokines in favor of pro-inflammatory ones leads to insulin resistance, systematic inflammation, oxidative stress, atherothrombosis and eventually, CVDs (28). However, systemic inflammation probably involves a complex network of signals interconnecting several organs. The causal relationship between systemic inflammation and adipose tissue inflammation is currently controversial. In summary, ATD increases cardiovascular risk through adipose tissue ectopic accumulation and dysregulation of adipokines in adipose tissue.

## 3 Gut Microbiota and Metabolites

### 3.1 Gut Microbiota—an Endocrine Organ

The term gut microbiota describes the commensal microbial species in the gastrointestinal tract (29). Trillions of microbial cells including bacteria, fungi, archaea, and viruses are harbored in the human intestine, which act as an essential and complicated part of our healthy physiological ecosystem. With a bacterial load of more than 10^14^ (30), the genome carried by the gut microbiota outnumbers human genome by 100 times (31). More than 90% of the taxa of the gut microbiota in adults is constituted by two major bacterial phyla, Bacteroidetes or Firmicutes, with a lower abundance of Actinobacteria, Cyanobacteria, Fusobacteria, Proteobacteria, and Verrumicrobia (32). Collectively, these bacteria make up the most complex and diverse ecosystem in human gut. Although the composition of gut bacteria is remarkably similar at the phylum level and a core of bacterial genera is present in the majority of adult hosts, there is huge variability at a subspecies level. The human gut microbiome is also highly dynamic and can be dramatically altered by age, antibiotic use, host genetics, chronic dietary patterns, and other environmental exposures (31, 33, 34).

Gut microbiome plays a predominant role in training and maturation of the host immune system (35), vitamin synthesis, resistance to colonization by or overgrowth of pathogenic microorganisms, deconjugation of bile acids (BAs) (36), and energy harvest through fermentation of indigestible carbohydrates (37). With this huge microbiome, diversity at the subspecies level, it is not surprising that the gut microbiota has a significant influence on the biological activity of the host. Indeed, the gut microbiota plays an important role in the regulation of metabolic activities of the human body, which is mainly achieved through its metabolites (38).

The Merriam-Webster dictionary defines an endocrine organ as follows: “producing secretions that are distributed in the body by way of the bloodstream.” Besides endocrine organs in the conventional sense like the hypothalamus, pituitary gland, thyroid gland, and adipose tissue, the gut microbiome also fits this classic definition. But unlike host endocrine organs, which produce only a few key hormones, the gut microbial endocrine organ has the unique potential to produce hundreds if not thousands of humoral agents defined as metabolites of gut microbiota. These metabolites are sensed by highly selective host receptor systems that elicit diverse biological responses (39), and finally, alter the metabolic functions of distal organs.

### 3.2 Metabolites of Gut Microbiota

**Figure 2:**
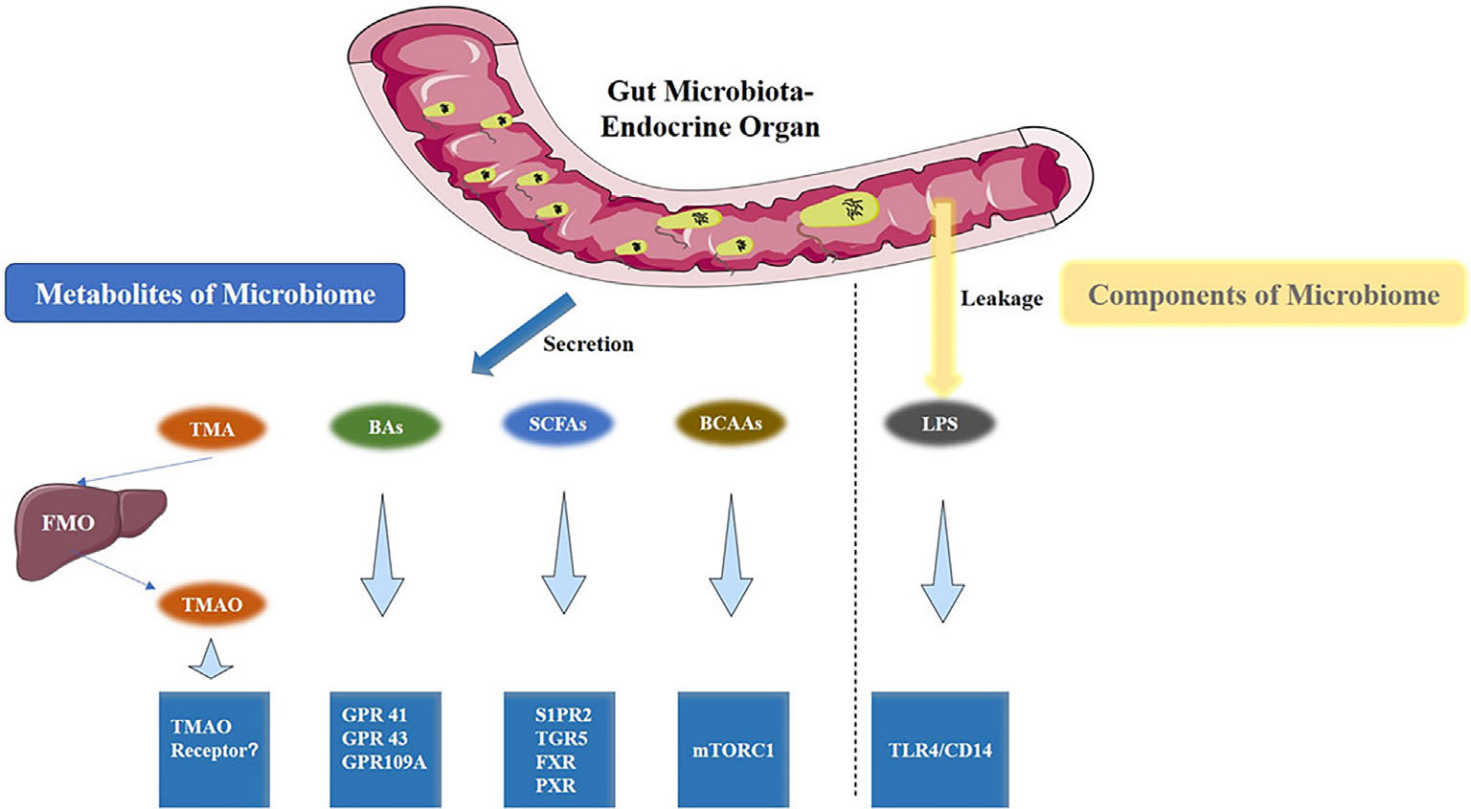
Gut microbiota metabolites and the associated metabolic signaling pathway. TMAO, BAs, SCFAs, BCAAs are the metabolites that are produced by the gut microbiota. Conversely, LPS is a component of the cell wall of gut bacteria. All are capable of activating specific signaling pathways. TMAO, Trimethylamine Oxide; BAs, bile acids; SCFAs, short-chain fatty acids; BCAAs, Branched-Chain Amino Acids; LPS, Lipopolysaccharides.

The gut microbiota produces a wide range of metabolites, such as vitamins and short-chain fatty acids (SCFAs), BAs, trimethylamine (TMA), branched-chain amino acids (BCAAs), ammonia, and phenols (8). These metabolites are absorbed by intestinal epithelial cell and finally enter the circulation. In addition, structure components of microbiome like lipopolysaccharide (LPS) could also enter the circulatory system of the host and become bioactive compounds (**Figure 2**). These microbiota-derived metabolites and LPS are agents of microbe-host communication, which is essential to maintain vital functions of the healthy host (39).

#### 3.2.1 Short-Chain Fatty Acids

SCFAs, also known as volatile fatty acids (VFA), are the end product of the gut microbiome’s fermentation of indigestible carbohydrates (such as fructose-oligosaccharides, inulin, oligosaccharides, non-starch polysaccharides, and oat bran) (40). SCFAs are a group of organic fatty acids with carbon chains between 1 and 6. They mainly include acetic acid, propionic acid, butyric acid, isobutyric acid, valeric acid, iso-valeric acid, iso-caproic acid, and hexanoic acid. The content of acetic acid, propionic acid and butyric acid is the highest in the intestinal tract: acetic acid (40–100 mmol/kg), propionic acid (15–40 mmol/kg), butyric acid (10–30 mmol/kg), the latter is the main SCFA in the intestinal tract (41). For healthy individuals, several factors affect the production of SCFAs, including the source and chemical properties of the substrate, the type and number of intestinal microorganisms, and the time of transportation in the intestine (42).

SCFAs have a variety of biochemical and physiological effects. First, SCFAs can be used for the biosynthesis of lipids, cholesterol, and proteins. Second, SCFAs act as signaling molecules to distant tissues and organs of the host. The effects of SCFAs are in part mediated by G-protein coupled receptors (GPR41, GPR43, and GPR109A) and histone deacetylase (HDAC), which are related to oxidative stress, the immune response, insulin resistance, and inflammatory responses (43, 44).

#### 3.2.2 Bile Acids

BAs are a general term for a large group of cholic acids that exist in the form of sodium salts or potassium salts. Primary BAs are synthesized from cholesterol in hepatocytes through multiple reactions such as hydroxylation, side chain oxidation, isomerization and hydrogenation, and finally forms chenodeoxycholic acid (CDCA) and cholic acid (CA). Further, primary BAs combine with glycine or taurine in the liver to form conjugated BAs, which enter the gallbladder. After food ingestion, the gallbladder releases these BAs into the duodenum, where they function in the small intestine and colon. After the BAs assist in the digestion of food, most are reabsorbed back into the liver in a process known as the enterohepatic circulation of BAs (45). A number of recent studies have found that unabsorbed BAs in the intestine can be used as substrates for microbial metabolism and further transformed into secondary BAs through hydrolase or dehydrogenase enzymes. These intestinal bacteria are mainly composed of *Lactobacillus, Streptococcus, Enterobacter, Enterococcus, Clostridium, Lactobacillus, Veronococcus* (46). They separate BAs from glycine or taurine to form mainly deoxycholic acid (DCA) and lithocholic acid (LCA), which in turn, may be further recirculated to the liver and, like primary BAs, combine with glycine or taurine and are excreted in small amounts in the feces (45).

BAs have many important physiological functions. Their function was first recognized as an emulsion to promote the absorption of fatty acids and fat-soluble vitamins in the human body. With their further study, BAs have attracted much attention as a signaling molecule for diverse endocrine and paracrine functions (47). BAs are able to bind to the G-protein coupled receptor sphingosine-1 phosphate receptor 2 (S1PR2) (48) and the Takeda G protein-coupled receptor-5 (TGR5) (49). BA can also activate farnesol nucleus receptor (FXR) (50), which induces fibroblast growth factor 15 (FGF15) expression and inhibits the expression of cholesterol 7α-hydroxylase (CYP7A1) in the liver, the rate-limiting step in BA synthesis, leading to decreased BA levels *via* a gut-microbiota-liver feedback loop. In addition, BAs have been reported to bind to vitamin D receptors and pregnane X receptors (PXR) *in vivo*. By activating these different signaling pathways, BAs play a role in regulating liver gluconeogenesis, glycogen synthesis, insulin sensitivity, and regulating the balance of energy metabolism in the body (51, 52).

#### 3.2.3 Trimethylamine-N-Oxide

Trimethylamine-N-Oxide (TMAO) is a monoamine metabolite found in gut microbiota that has attracted much attention because of its relationship with CVD in recent years (53). Foods containing choline, phosphatidylcholine, and L-carnitine are sources of TMA (54, 55). Choline, and its precursor phosphatidylcholine, are an abundant chemical constituents in daily diets such as animal liver, milk, and egg yolks. L-carnitine is an abundant nutrient in meat, especially red meats. Both choline and carnitine within the gut are absorbed within the small bowel *via* specific transporters, but absorption is incomplete, particularly with large meals that can saturate the uptake systems. Consequently, both dietary choline and carnitine ingestion can lead to significant elevations in TMA in the intestine. When TMA is absorbed into the circulation, it can be further delivery to the liver *via* the portal circulation, is rapidly converted into TMAO by hepatic flavin monooxygenase (FMO) (56).

Since TMAO was discovered, increasing research data, including human and animal models, has shown significant associations with a variety of diseases. Current studies have found that TMAO is closely associated with CVD (57), nephropathy (58), and diabetes (59). Although the relationship between TMAO and the above diseases has been established, the precise receptor or chemical sensor that detects TMAO remains unknown. Several possible mechanisms of TMAO in the occurrence and development of CVDs have been reported. Mechanisms mainly include (1): the inhibition of the reverse transport of cholesterol, and altering of the metabolic pathways of cholesterol and lipoprotein in the intestinal tract, blood vessel wall, liver, and other important organs (55) (2); promotion of vascular dysfunction and the inflammatory response through MAPK, NF-κB and NLRP3 inflammasome pathways (60, 61); and (3) the promotion of the release of Ca2+ in platelets, and then direct improvement of the response hypersensitivity of platelets, and acceleration of thrombosis (62).

#### 3.2.4 Lipopolysaccharides

LPS, known as a bacterial endotoxin, is a component of the outer membrane of Gram-negative bacteria and is important for maintaining the structural integrity of the bacteria. LPS consists of three components, including O-specific polysaccharides, core polysaccharides, and lipid A, which is the toxic center of LPS. Strictly speaking, LPS is not a metabolite of the gut microbiota. However, LPS still exerts its effects as a metabolism-independent signaling molecule. Dysfunction of the host’s gut such as following changes in permeability allows LPS to enter the circulation and activate several pathogenetic pathways (63).

First, LPS binds to the LPS binding protein (LBP), which transports LPS to the surface of immune cells and binds to the membrane protein receptor CD14. CD14 then transfers LPS to the Toll-like receptor 4 (TLR4) and myeloid differentiation protein 2 (MD2) protein complex. MD2 helps TLR4 recognize LPS, which activates intracellular signal transduction pathways and eventually activates the transcription factor NF-κB, generating pro-inflammatory cytokines such as TNF-α, IL-1b, and IL-6 (64).

#### 3.2.5 Branched-Chain Amino Acids

BCAAs, including leucine, isoleucine, and valine, are essential amino acids and BCAAs or BCAA-rich diets usually have a positive effect on body weight regulation, muscle protein synthesis, and glucose homeostasis (65). The gut microbiota plays an important role in both *in vivo* synthesis and absorption of BCAAs. *Prevotella copri* and *Bacteroides vulgatus* were found significantly associated with increased BCAAs biosynthesis and decreased transport of genes at the fecal metagenomics level. *Butyrivibrio crossotus* and *Eubacterium siraeum* were found to be associated with the uptake of BCAAs (66).

It is now well established that there is a very close relationship between BCAAs and insulin resistance. BCAAs are considered to be sensitive biomarkers in plasma that can respond to the degree of insulin resistance. However, it is unclear whether BCAAs are involved in the development of insulin resistance or whether they are just indicators of the disease (65). Current studies have assumed that BCAAs are anabolic signals that alter the growth of energy-consuming tissues, mediated in part through their ability to activate the mammalian target of rapamycin complex 1 (mTORC1) and protein kinase Cϵ (PKCϵ) (67), which may explain the bioactivity of BCAA in insulin resistance (67).

### 3.3 Techniques Used in Microbial Analysis

Although the identification methods of gut bacteria and their metabolites are not the focus of this review, a brief description of the common methods is necessary. Initially, bacterial culture was the only method to identify and analyze the gut microbiota (68), but with the advent of new techniques, it is current use is in the isolation and cultivation of a single bacterium, which is still important in establishing a link between bacteria and diseases (69). In addition, culture of single bacterium allows researchers to edit genes of a specific bacteria and to analyze its characteristics (70).

#### 3.3.1 Next Generation Sequencing

Emerging next generation sequencing (NGS) methods have replaced bacterial culture as the most efficient way to obtain information on all species of the human microbiome and even their respective genomes (71). To date, 2 common NGS strategies have been used in gut microbiota research: 16S ribosomal RNA (16S rRNA) gene sequencing and whole genomic shotgun sequencing (72).

Sequencing of 16S rRNA genes is currently the most widely used sequence-based technique for bacterial taxonomic and phylogenetic studies. It involves sequencing of specified microbial amplicons (mainly 16S rRNA). 16S rRNA genes are present in all gut microbiomes, encoding RNAs composed of small bacterial ribosomal subunits of the 30S. The16S variable regions are specific to bacteria and 16S rRNA gene sequencing has great potential for determining genera or species. Although 16S rRNA gene sequencing has important applications in bacterial identification and classification, its low resolution limits its application. In addition, 16S rRNA generally provides information about the composition of the microbiome, but lacks functional annotation (73).

Whole-genome shotgun sequencing means using high-throughput genome sequencing combined with advanced computational bioinformatics to identify taxonomic and potentially functional microbiomes. Theoretically it can bypass microbiome culture and identify the function of microbial communities, but it still requires more experimental confirmation (74).

#### 3.3.2 Mass Spectrometry Chromatography

In addition to the identification of the composition of the gut microbiota, the identification of the metabolites of the gut microbiota is equally very important. Mass spectrometry (MS), including tandem MS, is an important method for identifying metabolites of gut microbiota. Its high sensitivity allows us to analyze trace amounts of bacterial metabolites (75). MS is often coupled with chromatographic technology including gas chromatography (GC) and liquid chromatography (LC). This method combines the advantages of separation capabilities of chromatography and sensitivity of MS, reducing matrix effects and ion suppression that allows for a more accurate quantification and compound identification (76). This method is the most advanced technically, and most of the bacterial metabolites such as BA, SCFA, TMAO, LPS can be analyzed using this method (75, 77).

Furthermore, after identifying the metabolites of bacteria, functional omics techniques are used to link gut microbiota composition, metabolites, and diseases together. These techniques include transcriptomics, proteomics, and metabolomics (78).

## 4 Gut Microbiota in ATD Induced CVDs

### 4.1 “Gut Dysbiosis”

As mentioned above, although the gut microbiota is relatively stable in healthy individuals, it constantly changes throughout the life in response to endogenous and exogenous factors. From the moment intestinal bacteria are established in the infant, its composition and abundance are constantly modified under the influence of various factors such as delivery method, food, age, and exposure to antibiotics (79). Although the gut microbiota is constantly changing, its function in the maintenance of host homeostasis remains stable within a certain range in healthy humans.

‘Gut dysbiosis’ refers to a morbid change in the composition of the gut microbiota and may be caused by several factors such as diet, increased stress, inflammation, and antibiotic use (80). Although there is not a clear cause-and-effect relationship between this change and disease, dysbiosis can be observed in a wide range of diseases. A classic example of gut dysbiosis is the change in *Firmicutes/Bacteroidetes* ratio. As the two most abundant phylum of gut microbiota, changes in *Firmicutes/Bacteroidetes* ratio have been associated with a variety of diseases, such as obesity, gallstone disease, hypertension (81). For example, Emoto et al. reported a characteristic change in microbial composition of patients with coronary artery disease in which there was a significant increase in *Lactobacillales* (*Firmicutes*) and a decrease in *Bacteroidetes* (82). Currently, emerging evidence has shown that gut dysbiosis is able to disturb homeostatic functions of many metabolic activities through alteration of its community structure and metabolites (83). Thus, gut dysbiosis is considered a basic process of gut microbiota influencing disease development such as adipose tissue dysfunction (84), insulin resistance (66) and CVD.

**Figure 3:**
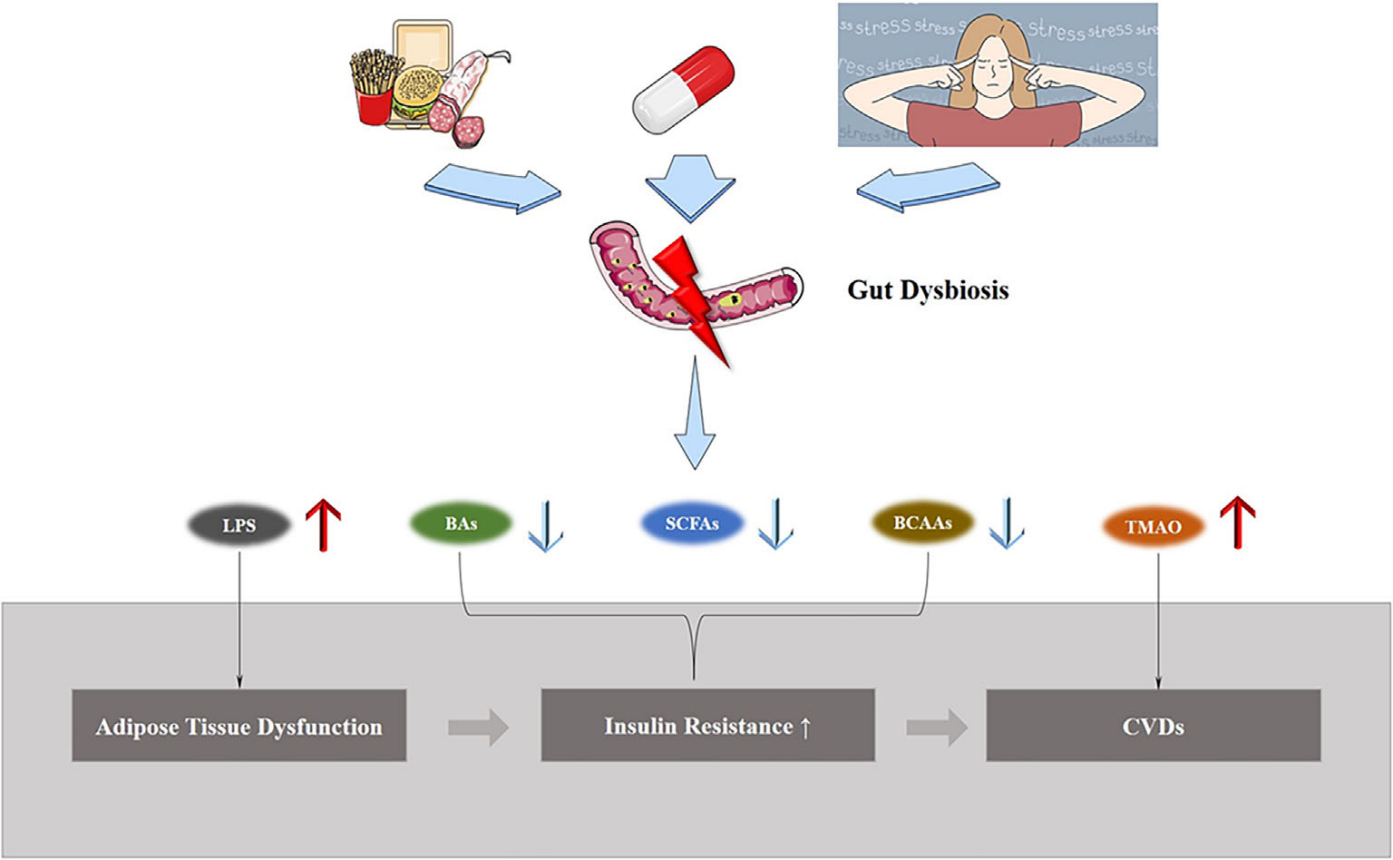
Gut dysbiosis and ATD induced CVDs. Exposure to external environment changes, such as a high-fat diet, treatment with antibiotics, and stress, lead to gut dysbiosis, causing changes in the structure of gut microbiota and metabolite levels, and increases the morbidity of ATD-induced CVDs.

However, gut dysbiosis involves alterations in a wide range of bacteria and their metabolites, so its relationship with adipose tissue dysfunction induced CVD is intertwined (**Figure 3**). Below we will explain the role of gut dysbiosis in the development of each aspect of the disease.

### 4.2 Gut Dysbiosis and Adipose Inflammation

The relationship between high-energy diet and obesity is unquestionable, and moreover, there is evidence that the intake of specific substances, such as saturated fatty acids (sFAs), induces adipose tissue dysfunction. With increasing understanding of the gut microbiota, scientists found that ATD is not purely a consequence of specific diet, but rather, may require the disturbance of intestine-microbiota interaction (85).

As mentioned, adipose inflammation is the most important feature in ATD. It is upstream of systemic inflammation and systemic insulin intolerance which leads to CVDs. Tran et al. (86) demonstrated that gut dysbiosis induced by a western diet is responsible for adipose inflammation in mice: an increase in classic proinflammatory M1 macrophages, and a decrease in anti-inflammatory M2 macrophages in adipose tissue were observed. Ablation of the gut microbiome could reduce such inflammatory responses in adipose tissue. Moreover, gene depletion of Toll-like receptor (TLR)-signaling adaptor protein myeloid differentiation primary response 88 (MyD88) largely phenotyped microbiota ablation, supporting the notion that western diet-induced adipose inflammation did not result from lipid accumulation per se, but rather was promoted by microbiota or their products activating innate immune signaling pathways. Although the study did not examine the metabolites of gut microbiota, it confirmed that gut microbiota and its metabolites induce adipose tissue inflammation through an inflammatory response related to TLRs. Previously, many researchers had long demonstrated that LPS was able to activate TLR (87) and LPS had been shown to be highly correlated with the development of obesity (88). The above findings could indicate a significant correlation between LPS produced by the gut microbiota and adipose tissue inflammation.

Moreover, recent studies are more supportive of the concept that dysbiosis of gut microbiota is an early event in inflammation and the development of obesity (84). When eating a high-fat diet (HFD), the first to be exposed to these nutrients is gastrointestinal system (89). A HFD induces gut dysbiosis and alters the secretory patterns of gut peptides. These changes can provoke an increase in the intestine mucosal inflammatory response, a disruption of the epithelial barrier, and a consequent enhanced transit of LPS into the systemic circulation.

LPS, as well as sFA consequent to a HFD intake may act in synergy to promote a harmful proinflammatory response as they are recognized as endogenous ligands for specialized TLRs, normally activated following the recognition of microbial-associated molecular patterns (MAMPS) by macrophages or intestinal epithelial cells (90), leading to a systemic low-grade inflammation. In recent years, researchers have found that TLR can also be expressed in adipose tissue, and have also found TLR-bearing macrophages resident in adipose tissue (91). Upon activation of TLRs, macrophages in adipose tissue are induced to the M1 pro-inflammatory phenotype and subsequently generate various inflammatory mediators (92). Adipose tissue may also secrete pro-inflammatory adipokines such as TNF-α, IL-6, IL-8, and MCP-1 in a TLR-dependent manner in response to LPS (93, 94). On initiation, this inflammatory environment may further recruit new inflammatory cells (neutrophils, macrophages), thus magnifying the inflammatory response in adipose tissue. Overall, systemic low-grade inflammation can promote adipose inflammation, leading to ATD, which in turn can further exacerbate systemic inflammation and insulin resistance, ultimately leading to CVDs.

### 4.3 Gut Dysbiosis and Insulin Resistance

Insulin resistance refers to a condition where the pancreas produces insulin, while muscle, liver, and other cells fail to respond to insulin, which leads to glucose intolerance and eventually diabetes, which is the most important link between ATD and CVDs. In parallel to ATD, gut dysbiosis can also directly mediate insulin resistance. In humans with obesity, a 7-day treatment with vancomycin, an antibiotic against Gram-positive bacteria, can modulate the composition of the gut microbiota and decrease peripheral insulin sensitivity (95). Adjustment in the Firmicutes/Bacteroidetes ratio has been proposed in insulin-resistant patients by many researches (96). In addition, intestinal bacterial species producing SCFAs such as *Roseburia*, *Eubacterium halii*, and *Faecalibacterium prausnitzii* are generally decreased, while opportunistic pathogens such as *Lactobacilli gasseri*, *Streptococcus mutans*, and *Escherichia coli* are increased in subjects with T2D (97). Thus, the relationship between insulin resistance and changes in intestinal bacterial genera and metabolites, with a major focus on BCAAs and SCFAs, has been demonstrated in many studies.

As mentioned, BCAAs are well-known biomarkers of insulin resistance and predictors of incident diabetes and CVDs. The gut microbiota has a wide range of enzymatic functions that trigger BCAA biosynthesis and BCAA are also adsorbed and enter the human circulation. Pedersen et al. (66) analyzed the gut microbiota and metabolites of 219 individuals without diabetes using non-targeted metabolomics and microbial genomic approaches. BCAAs were analyzed as important markers of insulin resistance. In addition, *Prevotella copri* and *Bacteroides Vulgatus* were found significantly associated with increased BCAAs biosynthesis in insulin resistance patients. Another study (98) also found that *P. copri* was the strongest driver species for the positive association between microbial BCAAs biosynthesis in the gut and insulin resistance, and *P. copri* was significantly increased in individuals receiving a HFD. Interestingly, Kovatcheva-Datchar Petia et al. (99) reported a contrasting conclusion between glucose intolerance and *P. copri* using a different dietary regimen, high in fiber and low in fat. Nevertheless, there is reason to believe that diet-induced gut dysbiosis influences the development of insulin resistance through changes in BCAA composition.

SCFAs, such as butyrate, acetate and propionate, display beneficial effects on peripheral tissues, such as adipose tissue, liver tissue, and skeletal muscle tissue, leading to an improvement in insulin sensitivity (100). SCFAs, as ligands, bind to FFAR2 and FFAR3 receptors distributed on enteroendocrine L-cells and play a role in regulating blood glucose. FFAR2 promote the secretion of insulin, GLP-1, GIP, and PYY secretion, promote the release of growth hormone, and reduce insulin signaling in fat. As for FFAR3, it promotes the secretion of GLP-1 and PYY, promote gluconeogenesis in the intestine, and decrease insulin secretion (101). In addition, increased permeability is associated with translocation of bacteria and their cell wall components which triggers an inflammatory cascade that has been associated with insulin resistance. SCFAs appear to play an important role in maintaining the integrity of the intestinal epithelial barrier through regulation of tight junction proteins, thus protecting the host from insulin resistance (102). When gut dysbiosis occurs, the SCFA-producing microbiome tends to decline, resulting in weaker regulation of blood glucose and reduction of insulin resistance by SCFAs, which in turn increases the risk of insulin resistance and even diabetes in the host (97).

BAs are also involved in glucose metabolism. Tauroursodeoxycholic acid is reported to have the potential to improve liver and muscle insulin sensitivity in obese hosts (103). Whist glycoursodeoxycholic acid (GUDCA) has the potential for treating hyperglycemia (104). Sun et al. (105) revealed that ablation of the gut microbiome alleviates HFD-induced glucose intolerance, hepatic steatosis, and inflammation by modulating the key enzyme CYP7A1 in the alternative BA synthesis pathway in hamsters, which indicates the dysbiosis of gut microbiota may contribute to insulin resistance by altering BA metabolism.

### 4.4 Gut Dysbiosis and Cardiovascular Diseases

Significant data has accumulated on the relationship between gut dysbiosis and CVDs. Extensive studies have demonstrated the role of gut dysbiosis in different CVDs, such as atherosclerosis (106), hypertension (107), and thrombosis (62). Numerous clinical trials have also demonstrated that many alterations in intestinal bacteria are significantly associated with the development of CVDs. *Collinsella, Proteobacteria, Actinobacteria, Enterobacteriaceae, Lactobacillales, Escherichia coli*, and *Klebsiella spp*, as well as the *Firmicutes/Bateriodetes* ratio, which has been reported to increase in CVD, as well as Butyrate-producing bacteria such as *Roseburia intestinalis*, while *Faecalibacterium cf. prausnitzii* has been reported to decrease in CVDs (82, 108–111). Notably, in ATD and insulin resistance, there is a clear causal relationship between gut dysbiosis and disease risk or severity, that is, such dysbiosis is the cause of disease onset. However, in heart failure, a CVD disease, there is evidence that the development of heart failure is responsible for the dysbiosis of gut bacteria (112). The mechanism might be the edema of the intestinal wall caused by systemic congestion secondary to heart failure systemic congestion. In turn, the highly permeable intestinal mucosa impaired the intestinal barrier function, leading structural components of microbiota (majorly LPS) to enter the circulation and to stimulate host immune responses and vascular inflammation, which causes gut dysbiosis to aggravate heart failure (113). Clinical trials have proven that enhanced abundance of pathogenic microbial were isolated from fecal samples of HF patients, especially in those with right ventricle heart failure and impaired intestinal barrier function (114).

Another common CVD, atherosclerosis, is also associated with gut dysbiosis. Koren et al. first reported the changes of gut microbiota were associated with atherosclerosis using 16S rRNA genes to survey bacterial taxa (115). At that time, whether these changes represented only microbial taxa associated with coronary heart disease or whether these alterations were risk factors that could promote the development of coronary heart disease has not been fully determined. With more contemporary studies to be reported, Jie et al. (116) observed an increased abundance of *Enterobacteriaceae* and oral cavity-associated bacteria and relatively depleted butyrate-producing bacteria in patients with atherosclerotic CVD *versus* those in healthy control subjects, suggesting that gut dysbiosis may be associated with the development of atherosclerosis. To date, the bulk of evidence suggests that gut dysbiosis is a significant risk factor for atherosclerosis.

TMAO, which links gut dysbiosis to atherogenesis, is one of the most important discoveries of recent years in the study of gut microbiota metabolites. In 2011, Wang et al. (53) identified a strong relationship between TMAO and the risk of CVD risks in a cohort study of approximately 2000 patients with atherosclerosis using a non-targeted metabolomics approach. This study suggested that level of plasma TMAO was strongly associated with future development of major adverse cardiovascular events (death, myocardial infarction, and stroke) in atherosclerosis patients. The mechanisms involved in TMAO-induced atherosclerosis have been described in previous sections. Notably, the value of TMAO is currently focused on prognostic value of circulating TMAO levels. Wang et al. (117). confirmed that TMAO predicted major adverse cardiac events in a 3-year follow-up cohort, even in the presence of elevated levels of non-microbial metabolites. Furthermore, the prognostic value of TMAO was also observed in patients with heart failure (118), diabetes (59), peripheral artery disease (119), and chronic kidney disease (58) independently of traditional risk factors.

Other metabolites such as BAs and SCFAs can also exert an effect on CVDs, but the role they play is that of physical modulators. These affect CVDs by modulating the upstream metabolic processes, as mentioned above, ATD, and insulin resistance. This also suggests that gut dysbiosis, by producing pathogenic effects and metabolic disorders at multiple points, permeates the development of CVDs due to ATD.

## 5 Gut Microbiome-Based Therapeutics in ATD Induced CVDs

As research progresses, we discover that several traditional therapies are able to influence the distribution of the gut microbiota. Many pharmaceutical effects that are difficult to explain by conventional theories can also be explained by changes in the gut microbiota. In ATD-induced CVDs, several conventional therapies have proven to be strongly associated with the modulation of gut dysbiosis.

### 5.1 Diet as an Important Modulator of the Gut Microbiota

What we eat, no doubt, is associated with ATD, insulin resistance, and other metabolic diseases. A breadth of knowledge exists regarding the impact of dietary fat intake on metabolic diseases (120, 121). For instance, De Souza et al. reported that consumption of HFDs correlated with insulin resistance (122). Woods et al. reported that a high fat intake is associated with increased fat storage (123). The gut microbiota serves as a filter for the largest environmental exposure—the diet. There have been several studies attempting to explain the role of the gut microbiota in the diet causing ATD, insulin resistance, and CVDs. For instance, HFDs have been associated with low-grade systemic inflammation *via* increases in circulating microbially-derived LPS. In addition, HFDs have been linked to atherosclerosis through the microbial production of TMA from L-carnitine and phosphatidylcholine. All of these findings suggest that modification of food composition may be the best way to treat metabolic disorders induced by gut dysbiosis because of its simplicity and ease of implementation.

The effects of a diet rich in saturated fat is highly likely to induce an increase in body mass, liver triglyceride content, and insulin insensitivity than a diet rich in monounsaturated fat or polyunsaturated fat by increasing the proportion of Firmicutes *versus* Bacteroidetes in the gut (124). In addition, long-term exposure to diets rich in L-carnitine and phosphatidylcholine cause excessive production of TMAO and subsequent induction of CVDs (125). In turn, by adjusting the intake of these substances, including of sFAs and choline, exercising, as well as intermittent fasting, we are better able to adjust the risk of metabolic diseases and CVDs (126, 127).

In addition, the intake of substances such as fish oil and nondigestible oligosaccharides has been shown to reduce the risk of metabolic diseases induced by gut dysbiosis. Caesar et al. (128) found that mice consuming a fish oil diet were protected from inflammation mediated by gut dysbiosis. This effect may be correlated with enrichment of the gut microbial genera *Akkermansia* and *Lactobacillus*. In addition, a high-fish-oil diet was reported to prevent adiposity and modulate white adipose tissue inflammation pathways in mice (129). Nondigestible oligosaccharides act as “fertilizers” of the gut microbiota, enhancing the growth of beneficial commensal organisms (e.g., *Bifidobacterium* and *Lactobacillus* species) (130). Cani et al. (131) found that oligofructose increased intestinal *Bifidobacteria* in HFD mice and that endotoxemia was significantly negatively correlated with *bifidobacterial* species. They also showed a significant positive correlation between *bifidobacterial* species and improved glucose tolerance and normalized inflammatory tone in high-fat oligofructose-treated mice. Neyrinck et al. (132) showed that wheat arabinoxylans in the diet counteracted high fat diet-induced gut dysbiosis with an improvement of obesity and lipid-lowering effects. In addition, the positive effects of wheat arabinoxylans including hypocholesterolemia, anti-inflammatory activity, and anti-obesity, have been associated with changes in the gut microbiota. Thus, we propose that by modifying food composition and intake of probiotic agents, can we effectively decrease the risk of ATD induced CVDs.

### 5.2 Metformin Exerts Therapeutic Effects by Improving Gut Dysbiosis

Metformin is an agent commonly used in clinical practice to treat diabetes. Metformin has also been reported to correct ATD (133) and to improve prognosis of CVDs (134). Initially The antihyperglycemic effect of metformin was mainly attributed to the reduction of intrahepatic gluconeogenesis. However, as research continued, metformin was found to be inextricably linked to the biological activity of the intestine. Bailey et al. described elevated levels of metformin accumulating in the gut of diabetes patients that were 300 times that found in the plasma (135). Forslund et al. reported metformin as a key contributor to changes in the human gut microbiome composition in patients with diabetes (136). Currently, many mechanisms have been reported in metformin activity in the treatment of insulin resistance and diabetes *via* improvement of intestinal dysregulation.

First, metformin is able to shift the gut microbiota toward a SCFA-producing microbiome in T2D individuals (137). Relative abundance of more than 80 bacterial strains are altered by metformin compared to placebo, where most changes are observed in the Firmicutes and Proteobacteria phyla, leading to an increase in SCFAs. In addition, metformin was reported to enhance the abundance of *Akkermansia muciniphila* (138), a mucin-degrading bacterial strain, which has gained considerable attention because of the reduction in the abundance of this bacterium associated with obesity, insulin resistance, diabetes, and CVDs in rodents and humans (139). Second, metformin treatment increases the levels of the BA glycoursodeoxycholic acid by inhibiting *Bacteroides fragilis*, thus improving insulin resistance by activating the FXR pathway (104). Third, metformin treatment is able to increase the abundance of Lactobacillus in the upper small intestine and restore the expression and thus increase the glucose sensitivity of the sodium glucose cotransporter-1 (SGLT1)-dependent pathway, which lowered glucose production in rodents (140).

### 5.3 Bariatric Surgery Alters Gut Microbiota in Metabolic Disease Patients

Bariatic surgeries (BSs) such as Roux-en-Y gastric bypass (RYGB) or sleeve gastrectomy(SG) are increasingly being used and proven to be effective treatment for diabetes and morbid obesity (141). Evidence has shown that changes in the gut microbiota are believed to play a role in metabolic improvements after BS. However, due to the small sample size of studies of gut microbiota after BS in humans, changes in the composition of the microbiota have yielded very contradictory results (142). For example, the abundance of Firmicutes was reported to be decreased in two studies (143, 144) but increased in two others (145, 146). Besides, Murphy et al. described an increase in the microbial population for Bacteroidetes after SG and a decreased population for the same phylum after RYGB (145). Meanwhile, Aron-Wisnewsky et al. (147). reported GS failed to fully rescue decreased gut microbial gene richness induced by severe obesity, despite the improvement in microbial gene richness. In any case, these contradictory results at least suggest that GS is able to exert an impact on the composition and abundance of gut microbiota.

Interestingly, an observational line study of 13 patients undergoing RYGB suggested that changes in microbial functional potential are greater than changes in microbial species abundance of the gut microbiota (148). This result reminds us that besides studying compositional changes, it is more important to pay attention to function and even metabolomics output of gut microbiota. A recent meta-analysis suggested that BCAAs were significantly decreased after BS, whilst TMAO levels were elevated in post-operative measurements. Although these changes different from those we may expect, they also suggest that a change in the level of metabolites is associated with gut microbiota alterations after BS surgery. The mechanisms induced by such changes and the effects on the organism remain to be further explored.

## 6 Discussion

We are exposed to an enormous variety of microorganisms residing in our gut, ranging from bacteria, viruses, fungi, and archaea to phages and protozoa. The gut microbiome can modulate nutrient metabolism upon dietary intake and produces many metabolites that interact with the host in a variety of ways. In this review, we have summarized the current knowledge on the gut microbiota in relation to ATD-induced CVDs in order to broaden our understanding in this field and to move towards establishing clinical applications, which may include a better understanding of etiology, pathology, and personalized interventions.

Nevertheless, additional studies are needed to clarify a number of issues regarding the relationship between the gut microbiota and ATD-induced CVDs. First, many of the relationships between gut microbiota and ATD-induced CVDs are based on findings from animal studies. The compositional differences of gut microbial communities between humans and rodents make animal study findings not directly translatable to humans (149). Observations or studies in human populations are lacking. Secondly, current studies have simply confirmed the presence of altered endocrine properties of the gut microbiota and its metabolites in patients with ATD, but whether such change is the cause or the consequence of ATD is still undetermined. Thirdly, current novel therapies for gut dysbiosis such as fecal transplantation (FMT) not only offers great potential for the treatment of a wide array of diseases, but are also a good model to study the cause-effect relationship between gut microbiota and metabolic disorders in humans. To date, animal FMT studies have demonstrated that the gut microbiota may modulate host metabolic diseases such as obesity and insulin resistance (150). In addition to FMT, transplantation of single probiotic species such as *Lactobacillus rhamnosus GG* has been shown to improve associated CVDs at the animal level (151). It cannot be denied that these therapies are very promising, but there is a lack of evidence for their effectiveness and safety in the population. A TMA-lyase inhibitor called 3,3-dimethyl-1-butanol, which in microbial cell cultures and *in vivo* mouse models reduces TMA/TMAO production without compromising microbial cell survival is another promising therapeutic approach lacking clinical evidence (152). In addition, interactions between the gut microbiota and existing drugs for CVDs such as aspirin, clopidogrel, statins, and angiotensin inhibitors are also worth investigating. Therefore, future research is necessary to incorporate the wealth of information on gut microbiota into clinical decision pathways and personalized treatment.

## Author Contributions

XY and XZ declare equal contribution to this work. QT and YW conceived the task. XY, QH, SL, and ZS performed the review and collected original studies. XY and XZ wrote the first draft of the manuscript. YW and QT revised the manuscript. WY, XG, HY, and HX contributed to language editing and final revision. All authors contributed to the article and approved the submitted version.

## Funding

The project was supported by the National Natural Science Foundation of China (No. 81973290,82070362), Beijing Key Laboratory of Non-Clinical Drug Metabolism and PK/PD study (Z141102004414062), the Key Project of Beijing Natural Science Foundation (No. 7181007).

## Conflict of Interest

The authors declare that the research was conducted in the absence of any commercial or financial relationships that could be construed as a potential conflict of interest.

## Publisher’s Note

All claims expressed in this article are solely those of the authors and do not necessarily represent those of their affiliated organizations, or those of the publisher, the editors and the reviewers. Any product that may be evaluated in this article, or claim that may be made by its manufacturer, is not guaranteed or endorsed by the publisher.

## Abbreviations

ANGPTL2, Angiopoietin-Like Protein 2; ATD, Adipose Tissue Dysfunction; BAs, Bile Acids; BCAAs, Branched-Chain Amino Acids; BMI, Body Mass Index; BS, Bariatric Surgery; CA, Cholic Acid; CDCA, Chenodeoxycholic Acid; CVDs, Cardiovascular Diseases; CYP7A1, Cholesterol 7α-Hydroxylase; DCA, Deoxycholic Acid; FGF15, Fibroblast Growth Factor 15; FMO, Flavin Monooxygenase; FMT, Fecal Microbiota Transplantation; FXR, Farnesol Nucleus Receptor; GC, Gas Chromatography; GUDCA, Glycoursodeoxycholic Acid; HDAC, Histone Deacetylase; HFD, High-Fat Diet; IL, Interleukin; LBP, Lipopolysaccharide Binding Protein; LC, Liquid Chromatography; LCA, Lithocholic Acid; LPS, Lipopolysaccharide; MAMPs, Microbial-Associated Molecular Patterns; MD2, Myeloid Differentiation Protein 2; MS, Mass Spectrometry; mTORC1, mammalian Target Of Rapamycin Complex 1; MYD88, Myeloid Differentiation Primary Response 88; NGS, Next Generation Sequencing; NO, Nitric Oxide; PKCϵ, Protein Kinase Cϵ; PXR, Pregnane X Receptors; RBP-4, Retinol Binding Protein 4; RYGB, Roux-en-Y Gastric Bypass; S1PR2, Sphingosine-1 Phosphate Receptor 2; SAT, Subcutaneous Adipose Tissue; SCFAs, Short-Chain Fatty Acids; sFAs, saturated Fatty Acids; SG, Sleeve Gastrectomy; SGLT1, Sodium Glucose Cotransporter-1; T2D, Type 2 Diabetes; TGF-β, Transforming Growth Factor β; TGR5, Takeda G Protein-Coupled Receptor-5; TLR, Toll-Like Receptor; TMA, Trimethylamine; TMAO, Trimethylamine-N-Oxide; TNF-α, Tumor Necrosis Factor α; VAT, Visceral Adipose Tissue; VFA, Volatile Fatty Acids; 16S rRNA, 16S ribosomal RNA.
